# Medical Opinion and Movement

**Published:** 1911-07-29

**Authors:** 


### Medical Opinion and Movement.

### Acromegaly.

At a meeting of the Société de Biologie, Drs. Claude and Baudouin reported an interesting case of acromegaly in which there appears to have existed a veritable anarchy of the internal secreting glands. The patient was a woman who in 1885, at the age of twenty-five, developed a typical condition of acromegaly. The classic symptoms of acromegaly successively presented themselves: hypertrophy of the hands, feet, and face, cervico-dorsal kyphosis, macroglossia. For ten years the arterial pressure was raised, amounting to 26 cm. and 28 cm. of mercury. At the same time this patient showed all the signs of Basedow's disease, exophthalmos, tachycardia, tremors, and pulsating goitre. Towards the end she was affected with marked asthenia, confining her to her bed. The symptoms therefore suggested a complete anarchy of the internal secreting glands, the hypophysis, suprarenals, and thyroid all functioning in excess, while the suppression of menstruation suggested the cessation of ovarian function as the *primum movens* of the whole condition. At the autopsy, a voluminous tumour of the pituitary body was found, weighing 48 grammes. All the other internal secreting glands were hypertrophied and in a state of hypersecretion. The ovaries were atrophied. The hæmolymphatic organs (spleen, lymphatic glands, etc.) were also hypertrophied.

### Ætiology of Scarlet Fever.

In the *Deutsche Medizinische Wochenschrift* Dr. Bernhardt gives an account of his researches on the infecting virus of scarlatina by inoculations in monkeys. Acting on the supposition that the scarlatinal virus, like that of variola, has a special affinity for the epithelial tissues, he employed as inoculating material the thick white covering of the tongue before it peels and takes on the characteristic strawberry appearance. By inoculation of this material into monkeys he was able to produce, after a variable period of incubation, a typical attack of the disease, analogous in all respects to scarlet fever in the human subject. He obtained similar results by inoculation of serum from the lymphatic vessels of the skin and from an emulsion of the lymphatic glands from patients who succumbed to the disease. Inoculations from one monkey to another produced the same typical symptoms. He found further that a generalised infection could be produced by buccal contamination without any cutaneous lesion. According to his experiments the scarlatinal virus belongs to the group of virus that can pass through the filter.

### Chronic Articular Rheumatism.

At a meeting of the Académie des Sciences, Dr. J. Bergonié read an interesting paper on chronic articular rheumatism and its treatment. According to Bouchard this disease belongs to a group which he designates diseases by retardation of metabolism. Adopting this hypothesis, in order to determine the physiotherapeutic measures necessary to modify the processes of nutrition one must first know the condition of ordinary metabolism of these patients. This the author has endeavoured to do by estimating the respiratory exchanges under the ordinary conditions of diet, external temperature, clothing, work, etc., of these patients. As a result of these observations he finds that the processes of combustion in this disease are actually very much diminished. The amount of oxygen absorbed by a chronic rheumatic patient may be reduced to a little more than three hundred litres in the twenty-four hours, whereas in a normal individual it is more than five hundred litres. From this it may be estimated that the metabolism is reduced to three-fifths. The author advocates as a means of increasing the metabolic activity, the electric stimulation of all the muscles of the body, the articulations remaining at rest. Light baths he considers quite inefficacious.

### The Heat and Rheumatism.

One of the effects of the dry fine weather has been an outbreak of rheumatism, which, in the opinion of the medical informant, is "the worst for many years." The medical informant appears to have included "intercostal neuralgia" and "stiff neck." So-called intercostal neuralgia is a very indefinite disease, most commonly apparently a referred pain which may have various origins. We have known of pain which might have been described as intercostal neuralgia which was said to occur during every menstrual period. Stiff neck and lumbago probably, too, have no connection whatever with rheumatism. Yet although such pains can scarcely be called rheumatism it would be interesting to learn whether there is any foundation for the statement that the heat has increased true rheumatism. The fact is well recognised that rheumatic fever is most common in the autumn, which means presumably that some influences are at work in the hotter period of the year which lead to its appearance later. Apparently, also, rheumatism is more common after hot, dry summers than after those comparatively cold and wet. But though this is the case, statistics seem to show that a marked rise in the number of cases is not present until September or October. If the rise in the number of cases is already very evident, the heat must have produced its deleterious effect early. Although it appears to be true that rheumatism is most frequent after dry summers, the popular belief that the disease starts in wet weather no doubt possesses a measure of truth. Cold and wet probably predispose to attacks of the disease. Most medical men must be familiar with cases of rheumatic fever which have commenced shortly after the patient has been in a heavy shower of rain. If, however, rheumatism is already common, there can have been no such predisposing cause for attacks of the disease.

